# A Propensity Score Analysis of Early and Long-Term Outcomes of Retrograde Arterial Perfusion for Endoscopic and Minimally Invasive Heart Valve Surgery in Both Young and Elderly Patients

**DOI:** 10.3390/jcdd9020044

**Published:** 2022-01-28

**Authors:** Hind Elhassan, Abdelrahman Abdelbar, Rebecca Taylor, Grzegorz Laskawski, Palanikumar Saravanan, Andrew Knowles, Joseph Zacharias

**Affiliations:** Department of Cardiothoracic Surgery, Blackpool Teaching Hospitals NHS Foundation Trust, Whinney Heys Rd, Blackpool FY3 8NR, UK; a.abdelbar@nhs.net (A.A.); rebecca.taylor73@nhs.net (R.T.); grzegorz.laskawski@nhs.net (G.L.); dr.saravanan@nsh.net (P.S.); dr.knowles@nhs.net (A.K.); mr.zacharias@nhs.net (J.Z.)

**Keywords:** minimal invasive cardiac surgery, retrograde arterial perfusion, neurological outcome, elderly patients, propensity score analysis

## Abstract

(1) Background: Minimal invasive cardiac surgery via right anterolateral thoracotomy for heart valve surgery and other intracardiac procedures proven to have lower postoperative complications. We aim to compare the neurological complications and post-operative outcomes in two cohort groups as well as survival rates up to 5 years postoperatively; (2) Methodology: Retrospective observational study for patients who had minimally invasive cardiac valve surgery with retrograde femoral arterial perfusion between 2007 and 2021 (*n* = 596) and the categorized patients into two groups based on their age (≥70 years old and below 70). Propensity match analysis was conducted. The primary endpoint consisted of major postoperative complications and the secondary endpoint was the long-term survival rate. (3) Results: There was no difference between the two groups in terms of postoperative outcomes. Patients ≥ 70 years old had no increased risk for neurological complications (*p* = 0.75) compared with those below 70 years old. The mortality rate was also not significant between the two groups (*p* = 0.37) as well as the crude survival rates. (4) Conclusions: The use of retrograde femoral arterial perfusion in elderly patients is not associated with increased risk compared to the younger patients’ group for a spectrum of primary cardiac valve procedures. Hence, minimally invasive approaches could be offered to elderly patients who might benefit from it.

## 1. Introduction

Minimal invasive cardiac surgery (MICS) has become an acceptable approach for cardiac valve procedures [1]. Minimal invasive cardiac surgery compared with the conventional median sternotomy is proven to be more cosmetic and has lower postoperative complications in terms of post-operative pain, reduced bleeding and need for transfusions, atrial fibrillation (AF), chest tube drainage, duration of ventilation, intensive care unit (ICU) stay and duration of hospitalization as well as return to normal activity [2,3,4,5]. 

However, minimally invasive surgery often requires the use of retrograde arterial perfusion from the femoral vessels, the use of retrograde femoral perfusion in minimally invasive surgery has been debatable especially in elderly patients due to the potential risk of embolic stroke [6,7].

In this study, we aim to establish the safety of minimally invasive primary valve procedures in selected elderly patients with retrograde femoral arterial perfusion by examining post-operative in-hospital outcomes and long-term survival compared to their younger cohort. 

## 2. Materials and Methods

This is a retrospective observational study involving consecutive patients who had minimally invasive heart valve surgery between 2007 and 2021 (*n* = 596) and had retrograde femoral arterial perfusion. Patients who were included in the study had undergone primary valve procedures (aortic, mitral, or tricuspid) regardless of the etiology. We had compared the early, mid, and long-term postoperative outcomes of patients 70 years of age or over, with those who were under 70 years old. The primary endpoint was a composite of major postoperative complications. Secondary outcomes consist of long-term survival up to three years postoperatively.

Data were obtained from our prospectively collected departmental database. Mortality was defined as death before hospital discharge or within 30 days of the operation. Stroke was defined as any new, permanent neurological deficit or lasting for more than 24 h or any new lesion on imaging. Renal complications were defined as the need for hemodialysis or an elevated creatinine level (>200 mmol/L). Postoperative myocardial infarction was defined as the presence of a new Q wave or a reduction in R wave in two contiguous leads. Chest infections needing reintubation, ventilation failure, reintubation for other causes, and tracheostomy all counted as respiratory failure.

## 3. Surgical Technique 

Operations were carried out via right anterolateral mini-thoracotomy; the shin incision located along the breast contour or pectoralis muscles border for mitral and tricuspid valves procedures the chest approached through the third or fourth intercostal space and via the second or third intercostal space for aortic valve procedures. Mitral valve procedures were carried out endoscopically and aortic valve procedures were carried out under direct vision. Retrograde perfusion was performed through a small incision in the groin with the Seldinger technique cannulation of the femoral artery and vein. Myocardial protection was achieved either with endo-aortic balloon occlusion or using an external aortic clamp. All patients had preoperative computed tomography of the thorax abdomen and pelvis (CT TAP) before the operations and intraoperative transesophageal echocardiography (TEE) was carried out routinely to ensure that there is no evidence of grades IV/V atheroma anywhere along the aortoiliac axis. Carbon dioxide insufflation was used in all patients. Patients with underlying obstructive peripheral vascular disease (PVD) were not offered a minimally invasive approach. 

## 4. Data Analysis 

Categorical variables are given as a percentage and continuous variables (e.g., age, clamp time, euro score) are given as “median, [Q1, Q3]” rather than mean and standard deviation as they are all non-normal. Differences were tested using Wilcoxon rank tests for continuous variables, and either chi-squared tests or Fisher exact tests for categorical variables. Fisher’s tests are used when the group sizes are small. A difference is considered statistically significant with *p* < 0.05.

To reduce the bias induced by the age groups’ imbalance of baseline characteristics, propensity score matching is used to create a set of patients with more balanced pre-operative characteristics except for their age; half are aged under 70 years and a half are at least 70 years or older, but are matched regarding sex, previous hypertension, previous stroke, previous COPD (Chronic Obstructive Pulmonary Disease), dyspnea status, being diabetic, presence of atrial fibrillation and logistic Euro Score. The caliper (the maximum tolerated difference between matched patients, in terms of standard deviations) was tightened until the resulting matched set did not display any significant imbalance in any of the pre-operative covariates. 

Matching was conducted using a greedy “nearest neighbor” algorithm on propensity scores calculated from a logistic model, with a caliper 0.1 times the standard deviation of propensity scores and 0.2 times the standard deviation of logistic Euro Scores. Adult (age < 70 years) and elderly (age ≥ 70 years) patients are paired 1:1 and without replacement. The resulting matched set comprises 112 pairs (224 patients) and the balance of pre-operative characteristics of this set. 

## 5. Results 

### 5.1. Patient Characteristics and Operative Data 

All patients who underwent MICS from 2007 to 2021 following the same standard surgical technique were included in the study, of these 241 (40.4%) were 70 years of age or above and 355 (59.5%) were below 70 years old. The baseline characteristics for these patients were compared. Table 1 shows that pre-operative differences exist in the two groups of patients under consideration beyond their age: the older patients (aged at least 70 years) comprise fewer males, have a higher prevalence of previous hypertension, stroke, and atrial fibrillation, and exhibit worse levels of dyspnoea. This is summarised in a considerably higher logistic Euro Score for patients over 70 than patients under 70 years (median 7.0 vs. 2.4, *p* < 0.001). 

Operative data are reported in Table 2. between the two groups, cardiopulmonary bypass (CPB) time (*p* = 0.036) and aortic cross-clamp time (*p* < 0.001) were statistically significant between the two groups.

### 5.2. Risk-Adjusted In-Hospital Outcomes 

**Figure 1:**
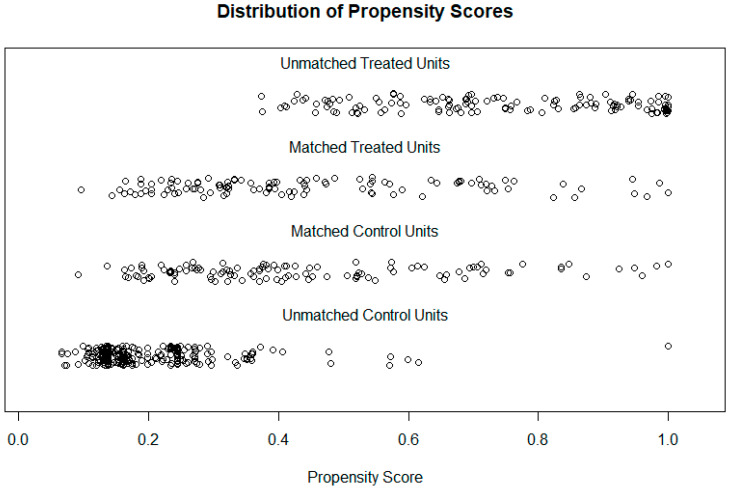
Jitter plot of propensity scores of matched and unmatched patients. “Treated” denotes elderly patients (age ≥ 70) and “Untreated” denotes adult patients (age < 70).

Figure 1 displays the distribution of propensity scores for the matched and unmatched sets; we see that the scores are similar for the matched pairs of elderly and adult patients and that the patients rejected had propensity scores much lower (“control”, age < 70) or much higher (“treated”, age ≥ 70) than those that were matched. This means that the matched set effectively contains patients in the “middle ground,” and conclusions drawn can only apply to patients in this range. 

Table 3 represents the resulting matched set which includes 112 pairs (224 patients) with balance preoperative characteristics. 

### 5.3. Primary Endpoint: Post-Operative Complications

Post-operative complications were reported in Table 4. There was no evidence of significant differences in any post-operative outcomes. Neurological complications (0.9% in elderly patients and 2.7% in the adult group) were insignificant between the two groups (*p* = 0.60). There was no reported myocardial infarction in the two groups and there was no difference in terms of ICU stay as well as hospital stay in the two groups (*p* = 0.77 and *p* = 0.38 respectively). The re-intervention rate for valve disease later in life was also insignificant in both groups (*p* = 0.60).

### 5.4. Secondary Endpoint: Survival Rates 

Survival rates for up to 5 years postoperatively had been calculated for up to 3 years postoperatively (Table 5) as comparison beyond this point is unreasonable to be linked to retrograde arterial perfusion as octogenarian patients by nature has more co-morbidities and more likely to die within 10 days compared to younger adult patients. 

The crude survival rates for one year and three years’ post-operatively are presented in Table 5. Figures suggest there were no significant differences in survival rates between age groups when the groups are similar in terms of baseline characteristics. 

The difference in restricted mean survival is not statistically significant at either 1 or 3 years but is almost significant by 5 years (Results in Table 6), where elderly patients have a mean survival of 0.29 years (around 3.5 months) lower than adult patients.

## 6. Discussion

Minimally invasive approaches have been increasingly popular over the past decade in primary heart valve surgery as well as other intracardiac procedures [8] with the use of retrograde arterial perfusion via femoral artery cannulation [1]. There are few studies that compared the outcome of minimally invasive mitral valve surgery to conventional sternotomy, and all had similar, or reported a favourable outcome for minimally invasive approaches [9,10,11] Many centers reported its reproducibility and safety [12,13]. The use of retrograde arterial perfusion has caused concerns as it could be associated with an increased risk of neurological complications, especially among elderly patients [14]. The literature search regarding this matter does provide controversial evidence. The Society of Thoracic Surgeons Adult Cardiac Database (STS ACSD) in 2010 reported double the risk of developing neurological complications following retrograde perfusion as compared to conventional sternotomy (*p* = 0.0002) [15]. However, this study has been criticized due to the lack of an accurate definition of MICS and there was no data on screening for PVD and aortoiliac disease [16]. The New York University (NYU) group reported the same finding of an increased risk of neurological complications associated with retrograde arterial perfusion (RAP) in patients with peripheral vascular disease or aortic disease (*p* = 0.001) [17]. The NYU group then emphasized the role of perioperative screening for peripheral and aortoiliac disease. Grossi et al. in the following paper reported the same finding but there was no significant neurological impact on those 50 years old or younger [6]. Cleveland Clinic group had screened low-risk patients before robotic MV (mitral valve) surgery for occult aortoiliac disease through contrast-enhanced multidetector CT [18]. Their study has identified asymptomatic patients with significant atherosclerosis. Both studies had reported that age and atherosclerosis were associated with increased risk for experiencing neurological complications [6,18]. Multiple confounding factors were identified with studies that reported higher stroke risk with RAP. The lack of screening for aortic atherosclerotic disease, the definition of the MICS procedure, retrospective observational studies that lack the reporting of PVD definition [16,19]. 

Minimal invasive cardiac surgery has been established in our hospital since 2007 using a right mini-thoracotomy with retrograde arterial perfusion through femoral artery cannulation with both endoaortic balloon occlusion and trans-thoracic aortic clamping. 

We aimed to compare the neurological complications and postoperative outcomes in two cohort groups, as well as the mortality rate and survival rates up to 5 years postoperatively. In our study, the compared postoperative outcomes in the two matched cohort groups were not statistically significant. We found that patients 70 years old or above had no increased risk for neurological complications compared with those below 70 years old (*p* = 0.60). This finding also applies to other major postoperative complications, as there was no increased risk of postoperative MI (*p* > 99), renal failure (*p* = 0.12), new AF (*p* = 0.66), pulmonary complications (*p* = 0.41) as well as GI complications (*p* = 0.68). The mortality rate was also not statistically significant between the two groups (*p* = 0.37). This result corresponds to the findings reported in the available literature [20,21]. 

The length of hospital stay was also not statistically significant (*p* = 0.38). The elderly patients’ group do get discharged home the same as the adult group rather than been referred for further rehab or repatriated to another hospital for recuperation (*p* = 0.42). Interestingly, the CPB time was significantly lower in the elderly group compared to the adult group (*p* = 0.036). This may be those particularly complex procedures were avoided in this age group. We have also looked at re-intervention procedures and only 1 patient from the elderly had redo for their primary value operation, while 3 from the adult group had redo operation at some point in their life (*p* = 0.60). 

Our strategy consists of screening patients for peripheral vascular and aortic disease through CT TAP and TOE. Extensive calcification of the arterial tree is not a contraindication to retrograde perfusion but any occlusive disease or the presence of irregular plaques within the femoral or iliac tree are reasons to avoid peripheral cannulation. We also avoid RAP for those with grade IV/V aortic atheroma picked up on the intraoperative Transesophageal echocardiogram. We routinely use CO_2_ insufflation in the surgical field during the procedure, which provides no increased risk in cerebral micro-emboli compared to conventional sternotomy [22].

There are a few limitations in this study that needed to be addressed. First, this is a single-center series, and the results may not be reproduced in other centers. We have a high population of elderly patients due to our location on the northwest coast of England. As we could not correct for age, we attempted to correct for other parameters to see if age would contribute to a higher risk of post-operative strokes and we hope this gives centers and surgeons starting a program some confidence in utilizing these techniques in the elderly. Secondly, as we have matched patients based on their baseline characteristics that means we have compared relatively healthier elderly to the adult group hence, the long-term outcomes for these patients cannot be generalized to elderly groups that are more ill. Furthermore, lack of a comparator group (elderly patients who had anterograde perfusion) we recommend further research in this aspect. Finally, the study has included all primary valve procedures regardless of the valve type (mitral, tricuspid, and aortic valve) which have different incision locations. 

## 7. Conclusions

In conclusion, from our institutional experience, the use of RAP during MICS for heart valve surgery was not associated with increased stroke incidence in those 70 years old and above compared to the younger group. The mortality rate and major postoperative outcomes were also similar for both groups. We conclude that the use of RAP via the femoral artery can be carried out safely for patients above 70 years of age who would benefit from minimally invasive heart valve surgery.

## Figures and Tables

**Table 1:** Patient characteristics (whole data set).

Variable	Elderly Patients Age ≥ 70 (*n* = 241)	Adult Patients Age < 70 (*n* = 355)	*p*-Value
Age	76.0 [72, 79]	58 [47, 65]	*p* < 0.001
Female	105 (43.6%)	116 (32.7%)	*p* = 0.009
BMI (Body Mass Index)	26.2 [23.3, 29.2]	26.5 [23.5, 29.5]	*p* = 0.41
DM (Diabetes Mellitus)	25 (10.3%)	21 (5.9%)	*p* = 0.065
HTN	148 (61.4%)	132 (37.8%)	*p* < 0.001
Peripheral vascular disease	13 (5.4%)	13 (3.7%)	*p* = 0.42
COPD	33 (13.7%)	50 (14.1%)	*p* = 0.97
CKD (chronic kidney disease)	4 (1.7%)	2 (0.6%)	0.37
Previous stroke: TIA (Transient Ischaemic Attack) CVA (cerebrovascular accident)	27 (11.2%) 9 (3.7%)	16 (4.5%) 12 (3.4%)	*p* = 0.008
Pre-op AF	55 (22.8%)	27 (7.6%)	*p* < 0.001
Urgent operation	16 (6.6%)	37 (10.4%)	*p* = 0.15
NYHA class III/IV	129 (57.5%)	151 (46.5%)	*p* = 0.011
Ejection fraction < 30%	6 (2.5%)	10 (2.8%)	*p* > 0.99
Logistic Euro Score	7.01 [4.25, 12.25]	2.4 [1.51, 4.38]	*p* < 0.001

HTN, hypertension; COPD, chronic obstructive pulmonary disease; AF, atrial fibrillation; NYHA, New York Heart Association classification

**Table 2:** Peri-operative information.

Variable	Elderly Patients >= 70	Adult Patients < 70	*p* Value
CPB time (mins)	146 [127, 172]	152 [131, 185]	*p* = 0.036
Aortic clamp time	96 [75, 111]	103 [82, 127]	*p* < 0.001
Mitral valve procedure +/− Tricuspid valve procedure +/− other	177 (73.4%)	291 (82%)	*p* = 0.017
Other cardiac procedure +/− valve	0 (0%)	23 (6.5%)	
Aortic valve procedure	64 (26.6%)	61 (17.2%)	*p* = 0.006
AF ablation	56 (23.2%)	40 (11.3%)	*p* < 0.001
Conversion to sternotomy	8 (3.3%)	16 (4.5%)	*p* = 0.61

CPB, cardiopulmonary bypass; AF, atrial fibrillation

**Table 3:** Patient characteristics (matched data set).

Variable	Elderly Patients Age ≥ 70 (*n* = 112)	Adult Patients Age < 70 (*n* = 112)	*p*-Value
Age	75.0 [71, 79]	64.0 [55, 68]	*p* < 0.001
Female	69 (38.4%)	57 (49.1%)	*p* = 0.14
BMI	26.4 [23.6, 29.2]	26.4 [23.2, 29.8]	*p* = 0.85
DM	6 (5.4%)	6 (5.4%)	*p* > 0.99
HTN	57 (50.9%)	57 (50.9%)	*p* > 0.99
Peripheral vascular disease	3 (2.7%)	8 (7.1%)	*p* = 0.22
COPD	14 (12.5%)	12 (10.7%)	*p* = 0.83
CKD	1 (0.9%)	2 (1.8%)	*p* > 0.99
Previous stroke: TIA CVA	9 (8.0%) 3 (2.7%)	8 (7.1%) 5 (4.5%)	*p* = 0.75
Pre-op AF	17 (15.2%)	17 (15.2%)	*p* > 0.99
Urgent operation	5 (4.5%)	11 (9.8%)	*p* = 0.19
NYHA class III/IV	55 (49.1%)	52 (46.4%)	*p* = 0.79
Ejection fraction < 30%	1 (0.9%)	7 (6.2%)	*p* = 0.07
Logistic Euro Score	4.83 [3.51, 7.46]	4.70 [3.19, 7.17]	*p* = 0.36

BMI, body mass index; DM, diabetes mellites; HTN, hypertension; COPD, chronic obstructive pulmonary disease; CKD, chronic kidney disease; CVA, cerebrovascular accident; AF, atrial fibrillation; NYHA, New York Heart Association Classification for dyspnea.

**Table 4:** Post-operative outcomes (matched set).

Variable	Elderly Patients Age ≥ 70 (*n* = 112)	Adult Patients Age < 70 (*n* = 112)	*p*-Value
Stroke: transient permanent	1 (0.9%) 2 (1.8%)	3 (2.7%) 2 (1.8%)	*p* = 0.60
MI	0	0	*p* > 0.99
New post-operative AF	6 (5.4%)	2 (1.8%)	*p* = 0.66
Renal failure	4 (3.6%)	0	*p* = 0.12
Re-operation (any purpose)	4 (3.6%)	4 (3.6%)	*p* > 0.99
Pulmonary complications	16 (14.3%)	11 (9.8%)	*p* = 0.41
ICU LOS (days)	1.0 [1.0, 1.0]	1.0 [1.0, 1.0]	*p* = 0.77
Inotropes	30 (26.8%)	34 (30.4%)	*p* = 0.66
GI complications	4 (3.6%)	2 (1.8%)	*p* = 0.68
Required blood transfusion	7 (7.1%)	6 (6.2%)	*p* = 0.87
Extubation > 12 h	10 (8.9%)	14 (12.5%)	*p* = 0.52
Duration of hospitalisation (days)	7 [5, 9]	6 [5, 8]	*p* = 0.38
Discharge destination (home)	105 (93.8%)	107 (95.5%)	*p* = 0.42
Reintervention	1 (0.9%)	3 (2.7%)	*p* = 0.60

MI, myocardial infarction; AF, atrial fibrillation; ICU LOS, intensive care unit length of stay; GI, gastrointestinal.

**Table 5:** Crude, unadjusted survival (matched dataset).

Variable	Elderly Patients Age ≥ 70	Adult Patients Age < 70	*p*-Value	The Relative Risk of Death for Elderly Patients over Adult (CI)
Mortality	4 (3.6%)	1 (0.9%)	*p* = 0.37	4.0 [0.45, 35.2]
One year survival	104/110 (94.5%)	99/101 (98.0%)	*p* = 0.28	2.8 [0.57, 13.3]
3 years survival	76/88 (86.4%)	85/90 (94.4%)	*p* = 0.078	1.7 [0.58, 5.0]

**Table 6:** Restricted mean survival times (RMST, matched set). Times are given in years.

Time Period (Postop)	Mean Restricted Survival in Years (95% Confidence Interval)		Difference in RMST
	Elderly (Age ≥ 70)	Adult (Age < 70)	
1 year	0.96 [0.92, 0.99]	0.98 [0.96, 1.00]	0.02 [−0.07, 0.02]
3 years	2.78 [2.65, 2.91]	2.90 [2.81, 2.99]	0.12 [−0.27, 0.04]
5 years	4.46 [4.21, 4.71]	4.75 [4.57, 4.93]	0.29 [−0.60, 0.02]

## Data Availability

The data presented in this study are available on request from the corresponding author. The data are not publicly available due to general data protection regulation in the UK.
